# Comparative Study between First and Second Harmonics of a Nd:YAG Laser for Cleaning Manifestation Damages That Appeared in Pigments Used on Archaeological Cartonnage

**DOI:** 10.3390/mi14071415

**Published:** 2023-07-13

**Authors:** Hala A. M. Afifi, Mona Abdel-Ghani, Raghda Mahmoud, Fatemah H. Alkallas, Amira Ben Gouider Trabelsi, Ayman M. Mostafa

**Affiliations:** 1 Conservation Department, Faculty of Archaeology, Cairo University, Giza 12613, Egypt; mabdelghan@yahoo.com; 2 Conservation Department, Grand Egyptian Museum, Cairo 11556, Egypt; raghdaeleraky@yahoo.com; 3 Department of Physics, College of Science, Princess Nourah Bint Abdulrahman University, P.O. Box 84428, Riyadh 11671, Saudi Arabia; fhalkallas@pnu.edu.sa (F.H.A.); aatrabelsi@pnu.edu.sa (A.B.G.T.); 4 Spectroscopy Department, Physics Research Institute, National Research Centre, 33 El Bohouth St. (Former El Tahrir St.), Dokki, Giza 12622, Egypt; 5 Department of Physics, College of Science, Qassim University, Buraydah 51452, Saudi Arabia

**Keywords:** Nd-YAG laser, XRF, optical properties, discoloration, magnetite, pigments, FT-IR, pollutant removal

## Abstract

This study focused on identifying the effect of the laser wavelengths used in cleaning some manifestation damage appearing in pigments used on archaeological cartonnage preserved in the Egyptian Museum, Egypt. The manifestations of damage appear as mud, resin, color, dust and microbiological damage stains. Lasers were chosen as one of the modern applications that give good results when cleaning the pigment materials without making direct contact with the material. Accordingly, lasers with a wavelength of 532 and 1064 nm were tested to identify their effect on stains caused by pigments and to choose the best one for use in cases similar to those materials in the future. This study was conducted to identify the effect of the selected wavelengths and choose the best ones to apply to the archaeological model. The evaluations were conducted using several tests and analyses, such as digital microscopy, X-ray florescence, Fourier transform infrared spectroscopy, and Handy colorimetry to evaluate that effect of lasers with a wavelength of 532 and 1064 nm to remove stains. The experimental study demonstrated the good effect of the Nd:YAG laser with a wavelength of 1064 nm compared with that of the 532 nm laser. The results of using the Nd:YAG laser proved the good effect of removing all stains compared with the 532 nm laser, which caused big changes when used to clean the stains on the pigment’s surfaces; it also did not help in removing or reducing some stains such as mud stains. According to these results, the good effect of the Nd:YAG laser (1064 nm) make it more suitable for cleaning than that of the Nd:YAG laser (532 nm), which is not recommended for use as it gave bad results when applied.

## 1. Introduction

Cleaning is usually the first step in any conservation project, depending on the condition of the object and the nature of the stains. When the decision is made to clean an object, it must be carried out with great consideration of the experiment to be performed to choose the proper cleaning method for the material composition in combination with the degradation phenomena [1,2,3]. Techniques based on the use of lasers have been used increasingly in archaeological fields and in conservation research and practice [3]. Lasers are considered one of the most important modern techniques used in the cleaning process, as they differ from the traditional cleaning methods, whether mechanical or chemical. The value of the laser technique is seen when it is used to clean pigment materials surfaces that are often very sensitive and difficult to clean, as the laser deals with the stains found on the surfaces of these materials without direct interaction by producing an intense laser beam in which each wave is coherent with the others near it [4,5,6]. One promising type of laser is the solid-state laser via YAG crystal, which is an acronym for neodymium-doped yttrium–aluminum–garnet (Nd:Y_3_Al_5_O_12_) [7,8,9,10], and emits typically in the near infrared region (NIR) at λ = 1064 nm (ω). Recently, this type of laser has been developed to emit radiation at λ = 532 nm (2ω), λ = 355 nm (3ω), λ = 266 nm (4ω), and λ = 213 nm (5ω) multiples of the fundamental wavelength using nonlinear crystals. It is used for restoration purposes [11] due to its relatively low cost, its availability and portability, the immediate control of the device, and the flexibility that the device provides in terms of pulses [12,13].

In the cleaning process, because the deposited pollutant layer on antique samples has a different thickness, the laser technique does not go beyond the sample layer of damage to the antique surface, which would cause damage to the antique, as it can be controlled by tunning the amount of energy, the duration of pulses, laser wavelengths, the number of pulses emitted each second, and the distance between the laser beam device and the surface to be cleaned. Tuning these parameter allows one to remove a specific amount of layers up to any required depth level with high precision, and the optional selectivity gives the restorer the ability to distinguish layers that will be removed while preserving the original surface [14,15,16,17]. This process depends on several factors, such as the surface reflection rate and the interconnectedness of its material, and the local application, where the area is cleaned to a direction only. Therefore, laser cleaning is considered a good choice for solving maintenance problems that cannot be solved by other methods or that require difficult solutions when using traditional cleaning methods. Also, it has the capability to treat the highly brittle effects that have altered objects, as it is possible to complete the cleaning process without mechanical pressure and thus without fragmentation or peeling [18,19]. In addition, when the laser radiation is released, the power of absorption and energy leads to rapid heating. This heating leads to the disintegration and expansion of dust, dirt, and corrosive particles so that they are easy to remove. From this standpoint, the use of Nd:YAG lasers could be considered one of the best solutions for cleaning pigmented archaeological materials because of its sensitivity.

A pigment, coming from the Latin pigmentum, meaning “drug”, is a finely divided and insoluble material that is suspended in a medium and acts as a coloring agent [20]. They were used widely during the ages of ancient Egypt in many fields such as wall painting, funerary art, and other arts. The use of pigments is considered one of the ways which religious, social, and environmental beliefs and symbols were expressed and are the most attractive targets for scientific study because their colors are yardsticks of a sense of beauty, and they provide a means for estimating ancient technologies’ ability to prepare pigments artificially [21,22]. The use of color in Egyptian paintings was highly symbolic and strictly regulated. Ancient Egyptian painters relied on six colors in their palette: red, which referred to life (a symbol of blood); green (a symbol to nature); blue (a symbol of life and birth associated with the annual flooding of the Nile and fertility); yellow (the color of the sun, gold, and a symbol of eternity); white (light); and black (a symbol of evil and darkness). Pigments were mixed with an organic binder as a medium such as gums or animal glue, which made them workable and fixed them to the surface being decorated [23].

Many researchers have studied the effect of using lasers on pigment materials [24] and have explained that the initial studies proved that the effect of lasers, whether Nd:YAG lasers, excimer lasers, or dye lasers, on various pigment materials varies due to the presence of organic media, as some wavelengths succeeded while others failed. For example, Pouli and Emmony et al. [25] indicated that vermilion, produced from HgS, always undergoes a color change when exposed to laser radiation at 0.3–0.5 J/cm^2^, which was supported by Abraham et al. [26], when he found that vermilion or HgS may undergo a color change that results from metamorphosis, which is a result of when the red-colored cinnabar is exposed to laser radiation, and its hex crystals turn into metacinnabar gray. Also, Chappé et al. [27] indicated that when lead red (Pb_3_O_4_) is exposed to laser radiation, a color change to gray occurs, and with an increase in the intensity of the laser energy, the color returns to orange, possibly due to a light–chemical reaction, which showed that chromium red (Pb(OH)_2_·PbCrO_4_) turns black when exposed to a laser wavelength of 248 nm and an influence 0.25 J/cm^2^ till 0.375 J/cm^2^; after that, the color was returned to its original one [28]. Furthermore, Chappé et al. [27] indicated that there is a relative change of Ukrainian structures (Fe_2_O_3_, Fe_2_O_3_·nH_2_O) to black color, depending on if the chosen laser wavelength is 532 nm or 1064 nm. In contrast, laser cleaning gave very good results when it was used to clean many colored materials. Sansonetti and Realini et al. [29] indicated that when white gypsum (CaSO_4_·2H_2_O) was exposed to the laser, it was found that it is very stable and does not show any color or morphological changes up to 3 J/cm^2^. In addition, they stated that when bone blacks (C) were exposed to lasers at 0.1 and 1 J/cm^2^, it was observed that no color or morphological changes occurred, as also indicated by Chappé et al. [27], who showed that cobalt blue (CoO·Al_2_O_3_) is relatively stable for lasers at short wavelengths and that the onset of its color change occurs at wavelengths of 1064, 532, 355, and 255 nm. Moreover, Bordalo et al. [30] also explained that when fermentation (Cu (C_2_H_3_O_2_)·H_2_O) was exposed to a laser, it was found that this color did not undergo any changes, confirmed by spectroscopic analysis, which showed that this color has no chemical changes. In addition, the 355 nm laser is the most efficient wavelength for removing soot and dust without damaging the original layers.

When archaeological cartonnage was buried, it experiences different types of damage pollution, such as mud, resin, color, dust, and microbiological damage stains. So, this work was focused on removing the dirt on the artifacts that was manually placed on the pigment layer, such as clay (mud), rosin resin, color, and dust. Then, the samples are placed in a humidifier containing distilled water and then displayed in a thermal oven.

1. Mud: It consists mainly of very small particles of alumina and silica bound together with water. These spots are produced as a result of the presence of cartons in a mud burial environment, which contributes to the appearance of mud spots.
2. Resin stains: These are caused by natural resin as well as an organic secretion that contains hydrocarbons from plants, especially pine trees. It is caused by the escalation of the materials used in embalming the mummy, causing stains on the carton covering the mummy.
3. Dust spots: The presence of dust and its accumulation on the carton’s surface is one of the main reasons for the formation of spots. Dust may include small particles and impurities that accumulate over time and depend on the preservation and storage conditions of the artifacts.
4. Microbiological damage stains: These may result from the presence of the carton in a display environment in which heat and humidity are not taken into account, or as a result of the presence of the carton in an unsuitable environment, such as in clay soil, which contributes to the emergence of fungal spores. These vital factors can interact with the chemicals included in the carton and cause damage to it, as well as lead to surface stains according to the type of fungus causing it. For example, *Asperigellus niger* causes black spots, while *Asperigellus flavus* causes green spots.
5. Color stains: These stains may be the result of the writings of kings in ancient times being distorted (for example, the king’s name was covered with them), which leads to damage and the staining of the surface and the colors it bears.

From this standpoint, in this research work, two wavelengths of the Nd:YAG laser system, the fundamental wavelength at 1064 nm and its second harmonic generation wavelength at 532 nm, were chosen to identify their effectiveness at removing stains from the surfaces of some colors that have not been adequately evaluated. The best laser was chosen for future applications.

## 2. Material and Methods

### 2.1. Preparation Method

**Figure 1:**
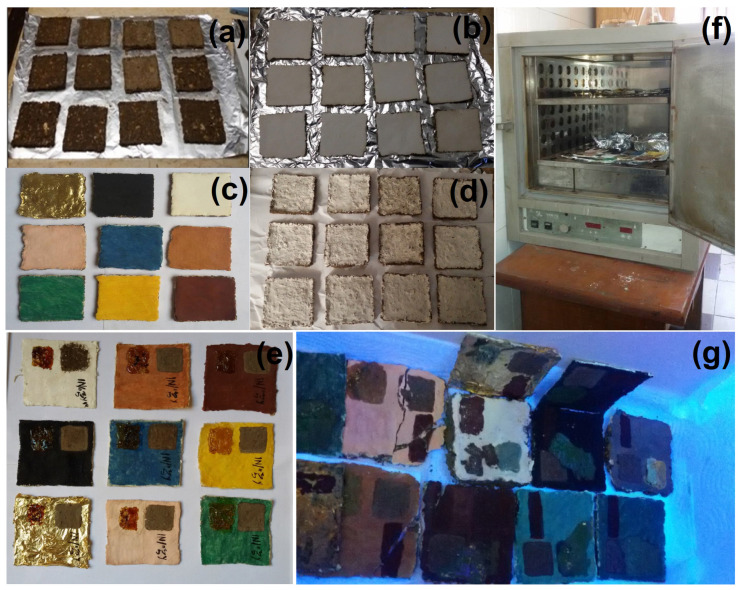
The prepared samples procedure: (**a**) the support layer consisting of linen or a mixed mud and sawdust; (**b**) the white preparation layer consisting of a calcium carbonate-covered support layer; (**c**) the applied paint layer above a layer of calcium carbonate; (**d**) the white finishing layer of calcium carbonate; (**e**) the addition of stains such as clay (mud), rosin resin, color, and dust; (**f**) the placement of samples in a thermal oven at a temperature of 80 ± 2 °C; and (**g**) the samples exposed to ultraviolet radiation for 48 h using fluorescent bulbs.

The studied samples were prepared in 7 × 7 cm^2^ squares for use in the experimental study, which are similar in cartonnage and chemical composition to the archaeological materials, consisting of linen or a mixed mud and sawdust (coarse type) as support. The samples were covered with a layer of calcium carbonate. After drying, the pigments were applied, including hematite, magnetite, Egyptian blue, orpiment, malachite, orange as a mixture of orpiment and hematite, calcite, and madder. Consequently, gold leaf was applied over an orange bole layer (goethite). Then, after drying the pigments, the samples were coated with a white preparation layer of calcium carbonate (Figure 1). The binding medium in the plaster layer, as well as the supporting polychrome layers, was identified as animal glue. After that, a colorimeter was used to measure the chromatic alterations in the samples. Then, visible dirt was manually placed on the pigment layer of the artifacts, such as clay (mud), rosin resin, color, and dust. Then, the samples were placed in a humidifier containing distilled water and then displayed in a thermal oven.

### 2.2. Characterization Techniques

The tests were carried out using a set of techniques, including digital microscopy (Dino-lite), X-ray florescence, Fourier transform infrared spectroscopy (FT-IR), and Handy colorimetry. These investigations indicate the nature of and damage to the pigment materials.

**Digital microscopy (Dino-lite):**

The surfaces of the samples were examined using Dino Capture 2.0, Version 1.5.12. It was used to recognize the surface’s forms of degradation such as stains and cracks using a magnification of 50×.

**Handy colorimetry:**

A precise color reader produced by Shenzhen Wave Optoelectronics Technology Co., Ltd., of the model WR-10QC, was used to determine the concentration of colored compounds for the standard sample and identify the changes resulting from the accelerated aging of samples, as well as identify the effects of the wavelengths used to clean the color.

**Fourier transform infrared spectroscopy (FT-IR):**

In this study, FTIR spectra of the paint media were obtained using a Bruker FTIR spectrometer, model VERTEX 70, equipped with ATR. The IR spectra, in absorbance mode, were obtained from the specimens using an aperture of 20–100 μm in the spectral region 600 to 4000 cm^−1^. The resolution was 4 cm^−1^, and the number of co-added scans was 64 for each spectrum.

**X-ray fluorescence (XRF):**

A portable XRF system (ELIO XRF spectrometer) from XGLAB (X and Gamma Ray Electronics) was used. It consists of a 50 kV Rh excitation tube, a Peltier-cooled silicon drift detector (with an energy resolution of 135 eV at the Mn K_α_ excitation line), and a set of pinhole collimators ranging from 2 mm down to 200 μm. The operating parameters for tube voltage and anode current during the measurements were set to 50 keV and 0.7 mA, respectively, and the real-time acquisition was 60 s. The diameter of the beam was set to 600 μm.

### 2.3. Accelerated Aging of Samples

Accelerated aging of samples was achieved according to Feller (1994), and the process began by using ovens at 80 °C, which rose progressively to 80 ± 2 °C. Then, samples were exposed to ultraviolet rays for 48 h using fluorescent bulbs (Figure 1).

### 2.4. Antimicrobial Investigation

**Figure 2:**
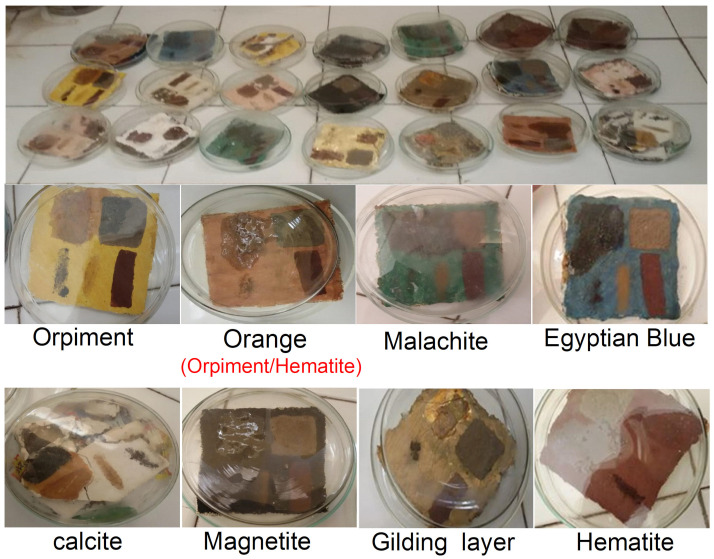
The growing of *Asperigellus niger* and *Asperigellus flavus* on experimental samples in Petri dishes and in a sterile environment.

**Figure 3:**
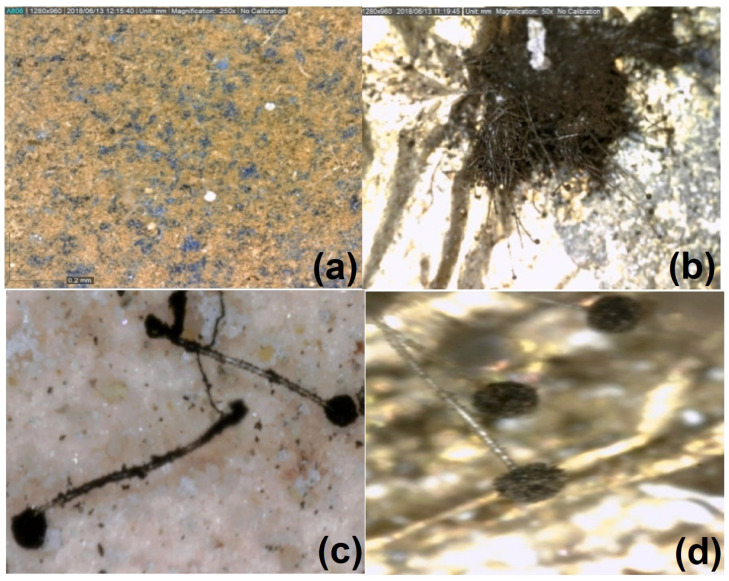
Digital microscope image (Dino-lite) with 250× magnification of (**a**) *Asperigellus flavus* on Egyptian blue, (**b**) *Asperigellus niger* on Egyptian blue, (**c**) *Asperigellus niger* on madder, and (**d**) *Asperigellus niger* on the gliding layer.

The antimicrobial studies were carried out to evaluate the antimicrobial behavior of the prepared composite. Cultures of the following microorganisms of the cartonnage samples were used in the tests: *Asperigellus flavus* and *Asperigellus niger* (Figure 2 and Figure 3).

### 2.5. Laser Cleaning

The equipment has cleaning and analyzing lasers. The specification of the used laser source is a Q-switched Nd: YAG Laser (PL9000, Continuum Electro-Optics, Inc. Laser, Boston, MA, USA), which emits a laser beam with a diameter of 1 cm, the energy of 200 mJ/pulse, a repetition rate of 15 Hz, a pulse duration of 7 ns, number of pulses set to 3, and fundamental wavelength of 1064 nm with a second harmonic generation of 532 nm. After that, the laser passes through the telescope structure consisting of a concave lens (f = 7 mm) and a convex lens (f = 7 mm) to correct the beam diameter to be 2 cm, which was suitable to cover the sample’s tested area in one shot. It was performed on a sample mounted on a holder, coupled to the X-Y-Z motorized stage, at atmospheric pressure.

## 3. Result and Discussions

### 3.1. Optical Digital Microscopy Investigation

**Figure 4:**
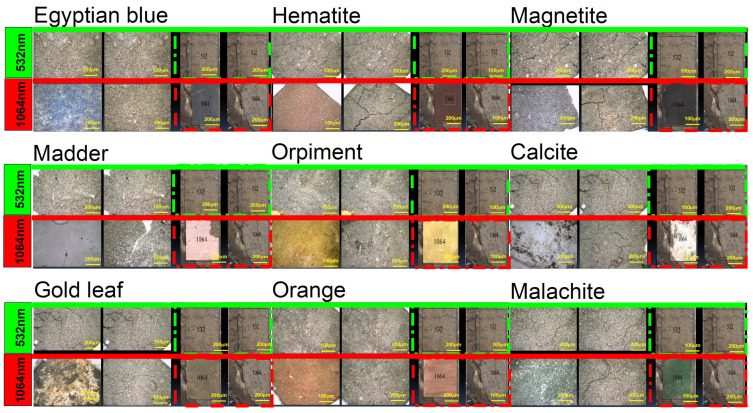
The effect of using lasers with 532 and 1064 nm wavelengths to clean clay stains on the paint layer under the digital microscope (USB).

**Figure 5:**
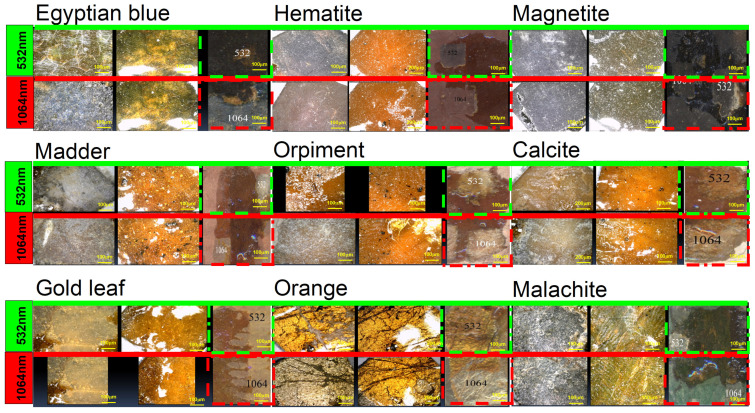
The effect of using lasers with 532 and 1064 nm wavelengths to clean resin stains on the paint layer under the digital microscope (USB).

**Figure 6:**
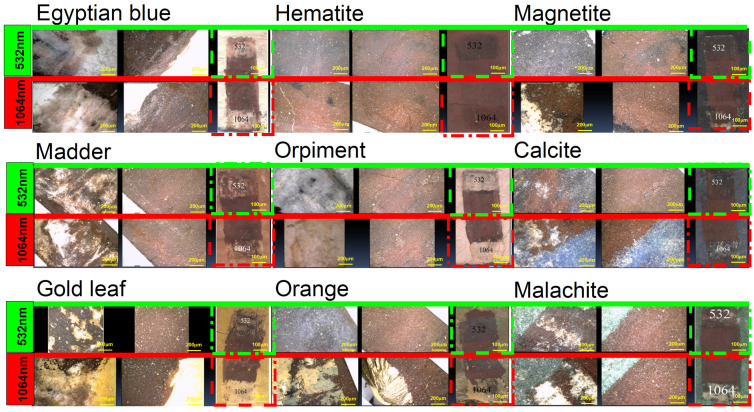
The effect of using lasers with 532 and 1064 nm wavelengths to clean color stains on the paint layer under the digital microscope (USB).

**Figure 7:**
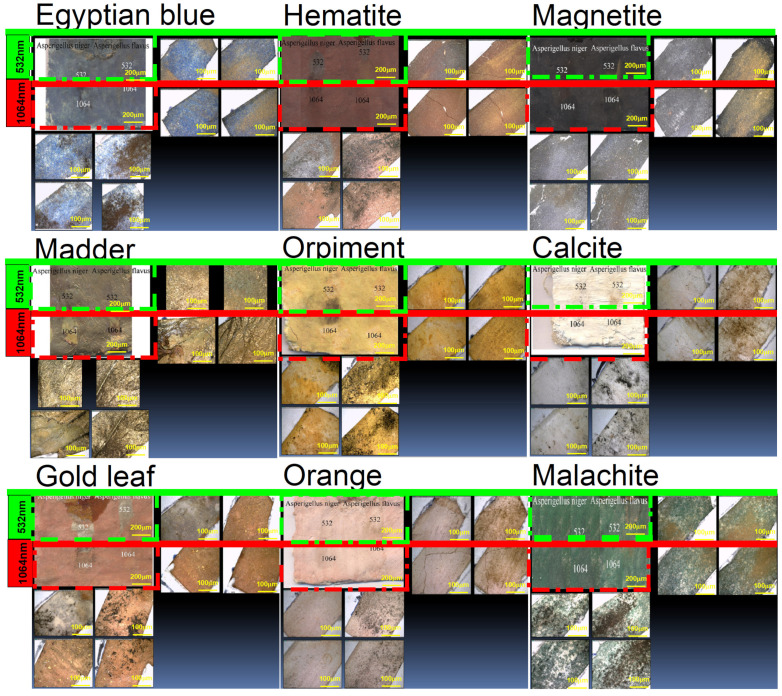
The effect of using lasers with 532 and 1064 nm wavelengths to clean microbiological damage such as *Asperigellus flavus* and *Asperigellus niger* on the paint layer under the digital microscope (USB).

**Figure 8:**
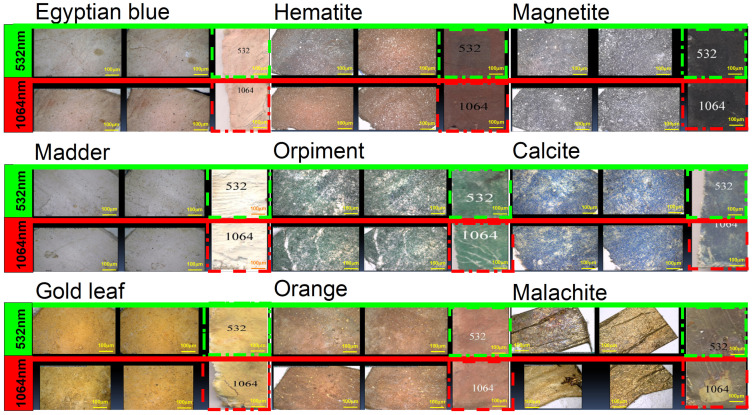
The effect of using lasers with 532 and 1064 nm wavelengths to clean dust stains on the paint layer under the digital microscope (USB).

In the present work, the effect of using lasers to clean the painting layer was studied. The painting layers consisted of hematite, magnetite, Egyptian blue, orpiment, malachite, orange as a mixture of orpiment and hematite, calcite, and madder, with gilding applied over an orange bole layer (goethite). The painting layers suffered from the appearance of some forms damage on their surface, such as clay (mud), rosin resin, color, microbiological damage, and dust stains, which may have resulted from the impact in an uncontrolled museum environment or from soil during extraction, which was not removed most of the time due to the weakness of the archaeological material. Another form damage of appeared: resin stains that may be produced intentionally or unintentionally as a result of the use of resin in embalming mummies. These forms are first cleaned mechanically under a digital microscope (USB) using cotton, wooden steaks, and chemical methods (using alcohol), which are sometimes not suitable if the painting layer is weak. This is why the lasers were used at 532 and 1064 nm. A comparison study was conducted using a digital microscope to clean mud (clay), dust, color, microbiological damage, and resin stains, which was carried out on clay (mud) stains and resin stains, as shown in Figure 4, Figure 5, Figure 6, Figure 7 and Figure 8.

In the case of clay (mud) stains, as shown in Figure 4, it is not preferable to use the 532 nm laser on the orange, hematite, madder, malachite, orpiment, calcite, Egyptian blue, and gilding layers because the clay stain remained the same without any benefit and did not give any result. It is preferable to use a laser with a wavelength of 1064 nm on the orange, hematite, madder, malachite, orpiment, calcite, Egyptian blue, and gilding layers, but in the beginning, mechanical cleaning with a wooden steak should be performed before laser cleaning. We found that if mechanical cleaning works to reduce the clay layer only and it is not completely removed, then lasers can be used to remove the remaining light layer of clay.

In the case of the resin stain, as shown in Figure 5, the orange color showed that the 1064 nm laser is preferred for cleaning because it gave an excellent result in removing the resin without any damage to the colored surface. However, it is not preferable to use a 532 nm laser for cleaning resin stains over the orange color because the laser removed the resin, the color layer, and the calcium carbonate preparation layer until it reached the support layer. On calcite and madder, it was shown that the use of a 532 nm laser works to partially break down the resin bonds, and it requires continued use of the laser, but this will waste time and the laser. As for the use of a 1064 nm laser, we find that it works to completely remove the resin, but a brown layer remains on the color, which is one of the resin residues. Therefore, the use of the laser is good, but a layer of resin remains. On hematite, it was shown that it is preferable to use the 1064 nm laser when cleaning the resin stain because it gives an excellent result in removing the resin without any damage to the colored surface. The 532 nm laser is not preferred for cleaning resin stains over hematite since it completely removes the resin but causes blackening. It is preferable to use the 1064 nm laser when cleaning the resin on the gilding layer because the laser works to completely remove the resin, giving an excellent result without any damage to the colored surface. The 532 nm laser is not preferred on the gilding layer because the resin spot remained the same, without any success. It is not preferable to use a 532 nm laser over malachite because the laser works to partially break the resin bonds, and by continually using the laser, it works to completely remove the resin, but it causes discoloration from light green to dark green. The 1064 nm laser is not preferred because it removes the resin completely, but causes discoloration from light green to dark green. On magnetite, it was shown that it is preferable to use the 1064 nm laser when cleaning resin stains because it gives excellent results in removing the resin without any damage to the colored surface. We find that using the 532 nm laser to clean resin stains on magnetite is good because it removes the resin and gives a very good result without causing any damage to the colored surface. It is not preferable to use either the 532 nm or the 1064 nm laser to clean resin stains over orpiment because both wavelengths work to remove the resin and the paint layer and a layer of calcium carbonate until it reaches the support layer. It was shown that that the 1064 nm laser is preferred to clean resin stains over Egyptian blue because it gave an excellent result in removing the resin without any damage to the colored surface. We find that the use of a 532 nm laser when cleaning resin stains on Egyptian blue only breaks the resin bonds without removing it, and with the continued use of the laser, it removes the resin completely.

In the case of color stains, as shown in Figure 6, it is not preferable to use a laser with a wavelength of 532 nm or 1064 nm to clean color stains on orange, malachite, and orpiment, as the laser beam cannot distinguish between colors, writings, and the paint layer as it works to remove the color stain until it reaches the calcium carbonate preparation layer and thus removes all of it. Its result is harmful to the sample. On calcite, it was shown that a laser that uses both colors to clean the color stain on white paint is good because it works to partially remove the red color stain. On madder, it was shown that using a 532 nm laser to clean the color stain is good because it partially removes the red color stain. However, the use of a laser with a wavelength of 1064 nm to clean the color stain is harmful because it works to clean the color stain, but the writing is removed because the laser beam cannot distinguish colors. It was found that a 532 nm laser in color stain on hematite is good, but it makes the material black. However, using a laser with a wavelength of 1064 nm to clean color stains on hematite is harmful because it cleans the color stain but removes the writing because the laser beam cannot distinguish the colors. The use of a 532 nm wavelength laser to clean color stains on gilding layer was shown to be good, but it blackens red color stains. It is not preferable to use a laser with a wavelength of 1064 nm to clean color stains on the gilding layer because it works to remove the color stain and the gilding layer down to the underlying layers because the laser beam cannot distinguish colors, and thus its result is harmful to the sample. On magnetite, it was shown that using a 532 nm laser to clean color stains is good, but it works to blacken red color stains without any removal effect, so the stain remains the same. It is not preferable to use a laser with a wavelength of 1064 nm to clean red color stains on magnetite because it works to remove the color stain, writings, and the black coloring layer down to the preparation layer of calcium carbonate because the laser beam cannot distinguish colors, and thus its result is harmful to the sample. It is not preferable to use a laser with a wavelength of 532 nm to clean red color stains on Egyptian blue because it blackens the blue color and only removes part of the color stain because the laser beam cannot distinguish colors. It is not advisable to use a laser with a wavelength of 1064 nm to clean color stains on Egyptian blue because it works to remove the color stain, writings, and the blue coloring layer down to the preparing layer of calcium carbonate because the laser beam cannot distinguish colors.

In the case of microbiological damage such as from *Asperigellus flavus* and *Asperigellus niger*, as shown Figure 7, it is preferable to use a laser with a wavelength of 1064 nm when cleaning fungus stains from *Asperigellus flavus* and *Asperigellus niger* over the orange color because it gives an excellent result in removing the fungus stains without any damage to the colored surface. It is not preferable to use the 532 nm laser when cleaning fungus stains from *Asperigellus flavus* over the orange color because it works to remove the fungus stain, paint layer, and preparation layer of calcium carbonate until it reaches to support layer. On calcite, madder, malachite, Egyptian blue, and magnetite, it was shown that lasers with both wavelengths are preferred for cleaning fungus stains from *Asperigellus flavus* and *Asperigellus niger* because they give excellent results in removing the fungus stains without any damage to the colored surface. On hematite, it is preferable to use the 1064 nm laser when cleaning *Asperigellus flavus* stain and *Asperigellus niger* stains because it gives an excellent result in removing the fungus stains without any damage to the colored surface. The 532 nm laser is not preferred when cleaning fungus stains from *Asperigellus flavus* and *Asperigellus niger* over hematite because it completely removes the fungus stains but blackens the hematite. On the gilding layer, it was shown that the 1064 nm wavelength laser is preferred for cleaning *Asperigellus flavus* stains and *Asperigellus niger* stains because it gives excellent results in removing the fungus stains without any damage to the colored surface. However, the 532 nm laser is not preferred for cleaning *Asperigellus flavus* stains and *Asperigellus niger* stain on the gilding layer because it creates a discoloration effect on the gilding layer, turning it to a reddish color, and completely removes it and affects the appearance of the orange bole layer (goethite). It is preferable to use a laser with a wavelength of 532 nm when cleaning fungus stains from *Asperigellus flavus* and *Asperigellus niger* over orpiment, as it gives an excellent result in removing the fungus stains without any damage to the colored surface. It is not preferable to use a laser with a wavelength of 1064 nm when cleaning fungus stains from *Asperigellus flavus* and *Asperigellus niger* over orpiment because it works to remove the fungus but also removes the orange bole layer (goethite) until it reaches the preparing layer of calcium carbonate.

In the case of dust stains, as shown in Figure 8, it is preferable to use the 1064 nm laser when cleaning the orange color and related dust because it gives an excellent result in removing the dust stains without any damage to the colored surface. It is not preferable to use a laser with a wavelength of 532 nm for cleaning orange color and related dust because it works by discoloring the color from orange to dark brown. It is preferable to use the lasers with a wavelength of 532 nm and 1064 nm when cleaning calcite, madder, malachite, Egyptian blue, and magnetite and their related dust, as they give excellent results in removing dirt stains without any damage to the colored surfaces. It was shown that the laser with a wavelength of 1064 nm is preferred for cleaning hematite and related dust, as it gives an excellent result in removing dust stains without any damage to the colored surface. However, the 532 nm laser is not preferred for cleaning hematite and related dust, as it works to blacken the hematite. It is preferable to use a laser with a wavelength of 1064 nm to clean the gilding layer and the dust associated with it because it provides an excellent result in removing dust stains without causing any damage to the colored surface. It is not preferable to use the 532 nm laser when cleaning the gilding layer and the dust associated with it, as it makes the gilding paper discolor to a reddish color. It is preferable to use the laser with a wavelength of 532 nm when cleaning orpiment and its related dust because it gives an excellent result in removing dust stains without any damage to the colored surface. It is not preferable to use the laser with a wavelength of 1064 nm when cleaning the orpiment and related dust because it causes discoloration from yellow to dark yellow.

### 3.2. Measurement of Color Change by Spectrophotometer

The measurement of the degree of a color change depends on the measurement of the color area of the material in an area of 1 cm^2^, which is the window area of the device, where the coordinates (a × b × L) are recorded under the color scale. The values of the change in L express the color’s niche from light to dark. The values of a × b express the color’s direction to the red region, where its decrease reflects the direction to the green area, while an increase of b expresses the direction to the yellow area, and its decrease expresses the direction to the blue region [31,32]. The measurements were made for the experimental aging samples after cleaning the stains on the surface of those pigments to identify the effect of the used wavelengths and to measure the total color change. The results are as follows.

**Figure 9:**
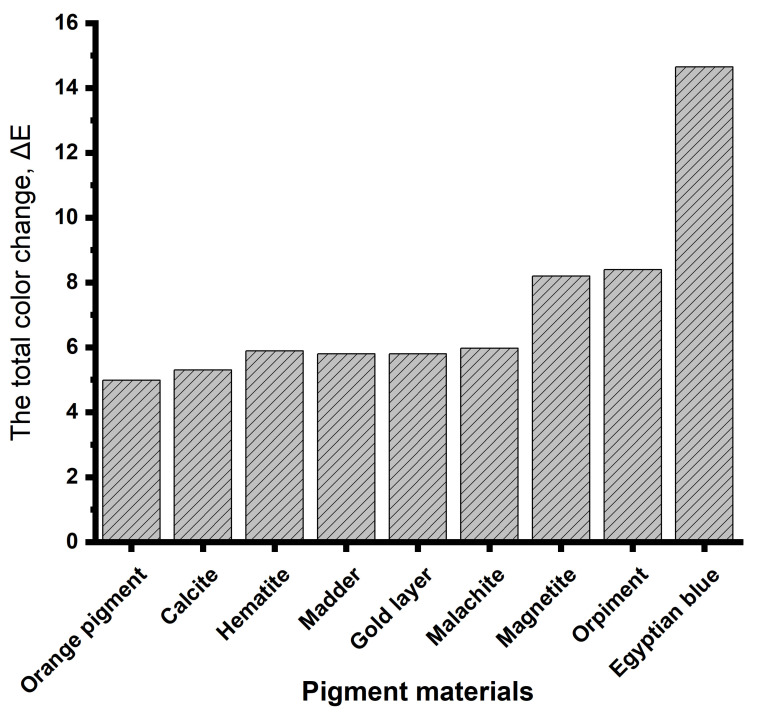
The total color change (ΔE) of the pigment materials under the effect of aging.

The results of the total color change of the samples, as shown in Figure 9 and Table 1, show the effect of artificial aging on the pigment materials. The results showed the total color change of the orange color, which gave a value equal to (ΔE = 4.98), while calcium carbonate gave a value equal to (E = 5.3), hematite gave a value equal to (ΔE = 5.89), madder gave a value equal to (ΔE = 5.8), the gilding paper gave a value equal to (ΔE = 5.8), and malachite gave a value equal to (ΔE = 5.97). Each of these colored materials (orange, calcium carbonate, hematite, malachite, madder and gilding paper) had a color change that can be seen with the naked eye. As for magnetite, it gave a value equal to (ΔE = 8.2), while orpiment gave a value equal to (ΔE = 8.4) and Egyptian blue gave a value equal to (ΔE = 14.65). Each of these colored materials (magnetite, orpiment, and Egyptian blue) experience great discoloration and distorted properties.

**Figure 10:**
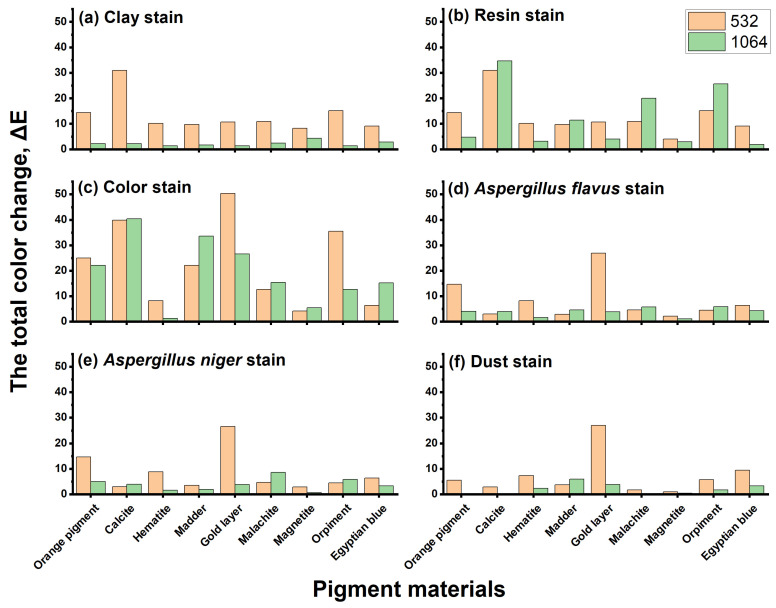
The total color change values for the samples (ΔE) before and after laser cleaning: (**a**) for clay stains, (**b**) for resin stans, (**c**) for color stains, (**d**) for microbiological stains (*Aspergillus flavus*), (**e**) for microbiological stains (*Aspergillus niger*), and (**f**) for dust stains.

By discussing the previous results, it becomes clear that Egyptian blue is the most damaged pigment material when exposed to aging, followed by orpiment, then magnetite. Meanwhile, the orange color had a color change that can be seen with the naked eye, followed by calcium carbonate, hematite, malachite, madder, and the gilding paper. Also, the color change results after cleaning the stains on the surface of the samples using the wavelengths used in this study are tabulated in S1:S6 and appear in Figure 10.

In the case of cleaning clay stains, the color changes in the pigment samples that were cleaned with the 532 nm laser are greater than those that were cleaned with the 1064 nm laser. In addition, we found severe visual distortions in the samples, and the use of the 1064 nm laser helped to remove the clay stains on the almost colored pigment and had no discernible color change, but it caused a slight color change on magnetite that can be observed with the naked eye.

In the case of cleaning resin stains, some color changes in the pigment samples before and after laser cleaning on the resin area were seen on the colored materials when using the 532 nm and 1064 nm lasers. From these results, it is clear that the use of the 532 nm laser in removing the resin stain on magnetite resulted in a slight color change that is noticeable to the naked eye, similar to the other pigment materials with severe visual distortions of the samples. However, the use of a 1064 nm laser to remove the resin stain on magnetite and Egyptian blue resulted in no discernible color change. As for the other pigment materials, such as orange, hematite, and gilding paper, they had slight color changes that can be seen with the naked eye. As for the other coloring materials, such as calcium carbonate, madder, malachite, and orpiment, we found severe visual distortions in the samples.

In the case of cleaning color stains, the samples before and after laser cleaning of the color stain showed the appearance of some color changes when using the 532 nm and 1064 nm lasers. It is evident that the use of a 532 nm laser to remove the color stain on magnetite resulted in a slight color change, but it is noticeable to the naked eye associated with severe visual distortions in the samples. Meanwhile, using the 1064 nm laser to remove the color spot on hematite, no discernible color changes occurred associated with many severe visual distortions.

In the case of cleaning microbiological stains (*Aspergillus flavus*), the samples before and after laser cleaning on the stain of *Aspergillus flavus* show the presence of color changes when using the 532 nm and 1064 nm lasers. It becomes clear that the use of the 532 nm laser to remove *Aspergillus flavus* fungus stains on calcium carbonate resulted in color changes, while the magnetite did not have a noticeable color change; for the other colored materials (orange, hematite, gilding paper, and Egyptian blue), we found severe visual distortions in the samples. In other words, by using a 1064 nm laser to remove the stains from the fungus *Aspergillus flavus*, there was no noticeable change in hematite and magnetite that can be seen with the naked eye.

In the case of cleaning microbiological stains (*Aspergellus niger*), the results of the total color change of the samples appear before and after laser cleaning of the stain from the fungus *Asperigellus niger* using 532 nm and 164 nm lasers. It is clear that the use of a 532 nm laser to remove *Asperigellus niger* fungus stains on calcium carbonate and magnetite did not result in significant color changes, while the other colored materials such as foah, malachite, and orpiment had a slight color change which can be seen with the naked eye. For other colored materials such as orange, hematite, gilding paper, and Egyptian blue, it severe visual distortions were found in the samples. In other words, using a 1064 nm laser to remove the stain from *Asperigellus niger* on hematite, the nozzle, and magnetite, these materials did not experience significant color changes. Other colored materials, namely orange, calcium carbonate, gilding paper, orpiment, and Egyptian blue, had a slight color change that can be seen with the naked eye. As for the remaining colored materials, such as malachite, we find severe visual distortions in the samples.

In the case of cleaning dust stains, the total color changes in the samples appear before and after the laser cleaning on the paint layer after cleaning with 532 nm and 1064 nm lasers. It is clear that using the 532 nm laser on calcium carbonate, malachite, and magnetite did not result in significant color changes, while other coloring materials, such as orange, madder, and orpiment, had a slight color change that can be seen with the naked eye. In addition, colored materials such as gilding paper, hematite, and Egyptian blue showed severe visual distortions in the samples. When using the 1064 nm laser with orange, calcium carbonate, hematite, malachite, magnetite, and orpiment, they did not experience significant color change. In addition, the other colored materials, such as madder, gilding paper, and Egyptian blue, had slight color changes that can be seen with the naked eye.

### 3.3. The Investigation of Functional Groups’ Pigments

**Figure 11:**
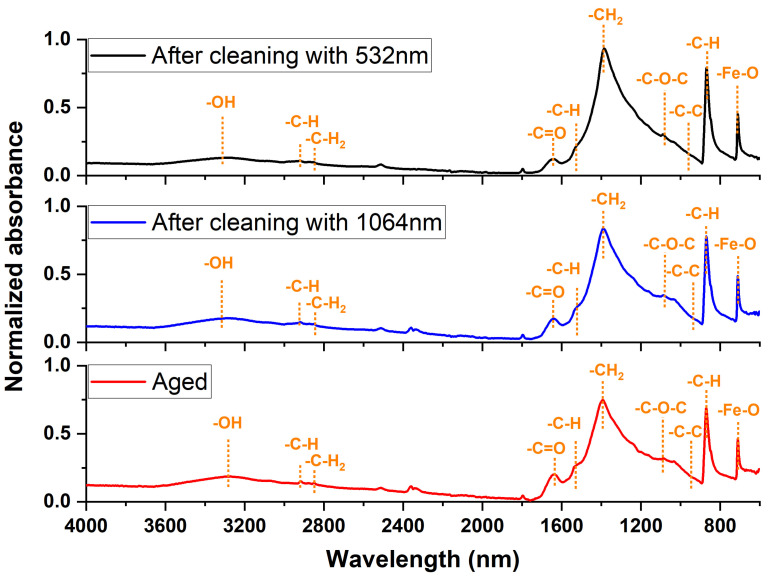
The absorption spectra of hematite before and after using lasers at 532 nm and 1064 nm wavelengths for cleaning dust stains.

FT-IR spectroscopic vibrational studies of the hematite sample (example of paint layer) before and after cleaning with 1064 nm and 532 nm pulsed lasers were conducted and shown in Figure 11. Looking at the vibrational motion of hematite before cleaning, the studied hematite sample showed the presence of the following functional groups: the hydroxyl functional group appeared in stretching mode with broad band peak around 3354 cm^−1^ and in bending mode with narrow peak at 1639 cm^−1^; CH and CH_2_ functional groups appeared in stretching mode at 2920 and 2850 cm^−1^; the C-C functional group appeared in stretching mode at 922 cm^−1^; the out-of-plane C-H band appeared in bending mode at 895 and 789 cm^−1^; and the Fe-O functional group appeared in stretching mode at 660 cm^−1^. After that, analyzing the functional groups of the hematite sample (example of paint layer) cleaned using the 532 nm laser to remove dust stains, it was observed that the functional groups of animal glue mixed with calcium carbonate. Following a comparison of these shown spectra with those of a standard sample of animal glue or a standard sample of CaCO_3_, significant changes were observed in the functional groups of glue and calcium carbonate. Also, the main characteristic functional groups of hematite started to be reduced in their intensity. Similarly, using FT-IR to analyze the part of the hematite sample that was cleaned using a 1064 nm laser to remove dust stains, it was observed that the functional groups of animal glue mixed with calcium carbonate [33,34,35]. By comparing these shown spectra with those of a standard sample of animal glue or a standard sample of CaCO_3_, slight changes were observed in the functional groups of glue or calcium carbonate. Also, the main functional groups of hematite were not significantly affected in their intensity compared with the structure of standard hematite. These results were compatible with a previous work, which showed that the 1064 nm laser is more effective than the 532 nm laser in every color, exception for black. The use of these longer wavelength pulses is expected to increase the conduction of heat to the bulk material compared with nanosecond 532 nm laser pulses, which enhances the vaporization and re-deposition of the metal. So, higher cleaning efficiencies are achieved after cumulative laser treatments, with better results at the wavelengths of 1064 nm compared to 532 nm.

### 3.4. Elemental Investigation

An X-ray fluorescent device has been used in two directions. The first direction is to ensure that the pigments used in experimental samples are the same pigments used in archaeological cartonnage (the main XRF results are presented in Supplementary File—Figure S1).

**Figure 12:**
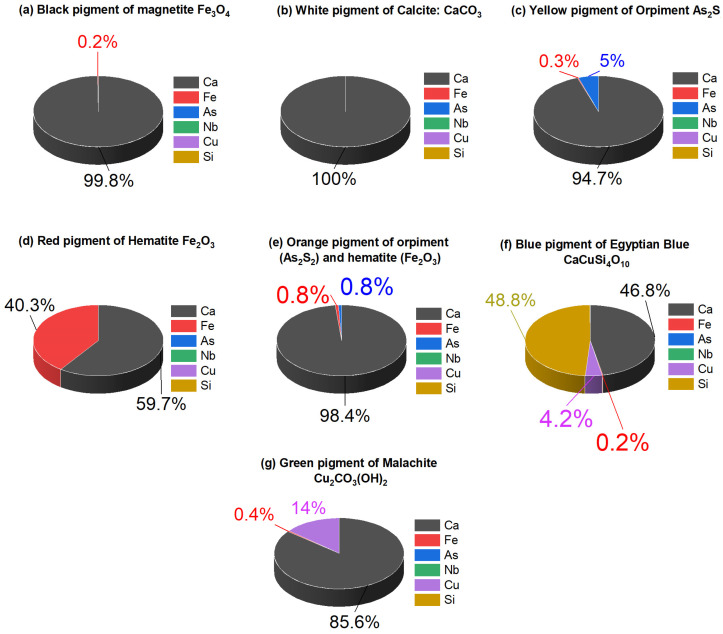
The pattern of analysis by X-ray fluorescence of (**a**) the black pigment of magnetite (Fe_3_O_4_), (**b**) white pigment of calcite (CaCO_3_), (**c**) the yellow pigment of orpiment (As_2_S_2_), (**d**) the red pigment of hematite (Fe_2_O_3_), (**e**) the orange pigment of orpiment (As_2_S_2_) and hematite (Fe_2_O_3_), (**f**) the blue pigment of Egyptian blue (CaCuSi_4_O_10_), and (**g**) the green pigment of malachite (Cu_2_CO_3_(OH)_2_).

For the black and white samples, the XRF spectra of the black paint (Figure 12a) exhibit iron (1%), suggesting the presence of the iron in the black pigment is magnetite (Fe_3_O_4_), and calcium (99%), which is a sign of the preparation layer. The XRF spectra of the white paint (Figure 12b) revealed the presence of calcium (100%), which confirmed the use of calcite. For the yellow paint sample, the XRF spectra of the yellow paint (Figure 12c) detected the presence of arsenic (5%) and sulfur (0.26%), which confirmed the yellow paint is orpiment (As_2_S_2_). Calcium (94.74%) was also found as a major element, most likely from the underlying preparation layer. For red and orange paint samples, the XRF analysis (Figure 12d) suggested the application iron (13.74%), which showed the use of hematite (Fe_2_O_3_) as a red pigment. Using XRF analysis (Figure 12e) in orange paint showed the presence of arsenic (1%) and iron (2%) that confirmed the orange paint is admixed with hematite and orpiment. In the red and the orange paint, a noticeable amount of calcite from the underlying plaster layer was also identified. For the blue and green paint sample, the XRF analysis (Figure 12f) confirmed the use of Egyptian blue (CaCuSi_4_O_10_), as copper (58.77%), silicon (4.18%), and calcium (36.88%) were detected. The XRF analysis (Figure 12g) of green paint confirmed the use of malachite (Cu_2_CO_3_(OH)_2_), as copper (4.99%) and calcium (94.59%) were detected. In blue and green paint, a noticeable amount of calcite from the underlying plaster layer was also identified.

**Figure 13:**
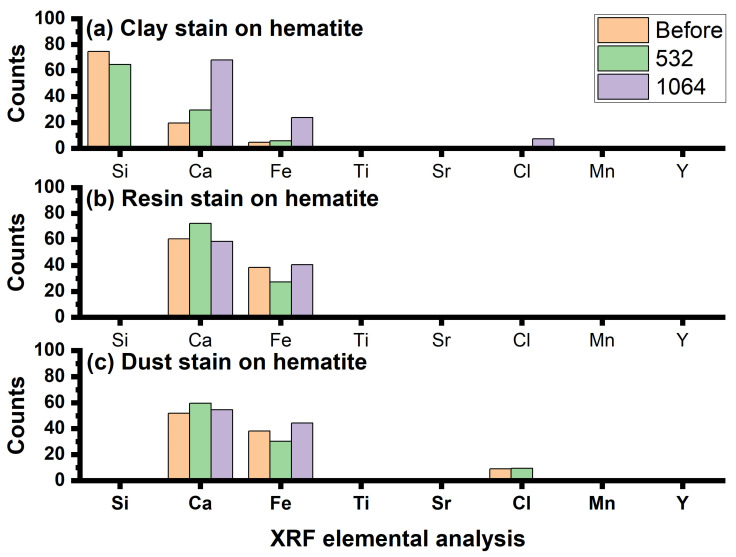
XRF analysis on the (**a**) clay, (**b**) resin, and (**c**) dust stains on hematite before and after using lasers for cleaning.

The second direction was based on making a comparison between the constituent elements of hematite pigments before and after cleaning different types of stains (clay, resin, and dust) using 532 and 1064 nm lasers on red hematite, which is represented by the graph in Figure 13 (the main results from the XRF data are presented in the Supplementary File—Figures S2–S4).

In the case of cleaning the clay stain, before using the laser, we found a high percentage of silica up to 74%, which is the main component of the clay stain, in addition to finding a decrease in the iron percentage of 4%, which is the main component of hematite, and a decrease in the percentage of calcium to 19%, which is the main component of the preparation layer. After using the 532 nm laser to clean the clay stain, we found a decrease in the percentage of silica, but not to a large degree, about 10%, and an increase in the percentage of iron to reach 5%, which is the main component of hematite, in addition to an increase in the percentage of calcium to reach 29%, which is the main component of the preparation layer. These results indicate that the effect was not desirable due to the fact that the 532 nm laser only reduces clay, but it is not completely removed. Also, after using the 1064 nm laser to clean clay stain, we observed the disappearance of silica and a big increase in the iron component by 23%, which is the main component of hematite, in addition to an increase in the percentage of calcium to 68%, which is the main component of the preparation layer; these results indicate that the 1064 nm laser has a proven ability to remove the clay stain completely and effectively [36,37].

In the case of cleaning resin stains, before using lasers to clean resin stains, we found that the percentage of iron was 38%, which is the main component of hematite. In addition to that, we find that the percentage of calcium is up to 60%, which is the main component of the preparation layer. After using the 532 nm laser to clean the resin stains, we found a decrease in the percentage of iron to 27%, which is the main component of hematite, in addition to an increase in the percentage of calcium to 72%, which is the main component of the preparation layer; these results indicate that the effect was not desirable because the 532 nm laser works to blacken and reduce the iron that forms the hematite. Also, after using the 1064 nm laser to clean the resin stains, we found a big increase in the percentage of iron to 40%, which is the main component of hematite, in addition to a decrease in the percentage of calcium to 58%, which is the main component of the preparation layer; these results indicate that the 1064 nm laser has a proven ability to remove the resin stain completely and effectively [38,39].

In the case of cleaning dust stains, before using lasers to clean the stains, we found that the percentage of iron reached 38%, which is the main component of hematite, in addition to increasing the percentage of calcium to reach 51%, which is the main component of the preparation layer. After using the 532 nm laser to clean the dust stains, we observed a decrease in the percentage of iron to 30%, which is the main component of hematite, in addition to an increase in the percentage of calcium to 59%, the main component of the preparation layer, which indicates that the result was not desirable because the 532 nm laser works to blacken and reduce the iron that forms hematite. Also, after using the 1064 nm laser to clean the dust stains, we find a large increase in the percentage of iron to 44%, which is the main component of hematite, in addition to a decrease in the percentage of calcium to reach 54%, which is the main component of the preparation layer, which indicates that the 1064 nm laser has proven its ability to remove dust stains from the red hematite.

Based on the results obtained in the present study, it was concluded that it is preferable to use a 1064 nm laser to remove the clay (mud), dust, resin, and microbiological stains. It gave very good results in terms of the degree of color change and the degree of cleaning compared to using the 532 nm laser.

The experimental study demonstrated the good effect of laser with a wavelength of 1064 nm, it did not result in noticeable discoloration nor any chemical changes to the most studied material. It also gave perfect results when used to clean most stains. It cleaned the stains without any damage to the pigment surface, whereas the 532 nm laser, when used in removing resin, damages the pigment layer (removing the pigment layer completely). When it was used to clean the resin stains on the surfaces of the pigments, the laser at 1064 nm gave excellent results on the pigment materials such as orange, hematite, magnetite, orpiment, calcite, Egyptian blue, and the gilding layer. It did not cause any noticeable color changes at the end of the process. With the exception of calcite and madder, which have a slight brown color on their surfaces, cleaning reveals the remainder of the resin layer, and a slight darkening occurs when used to clean malachite. By comparing these results with the results of cleaning resins from surfaces using the 532 nm laser, the tests proved that the 532 nm laser gave very bad results when used on gilded surfaces, as it did not give noticeable results to remove the resin and did not give good results when cleaning the orange color, as it removes the resin and the color layer together as it operates. It breaks the resin bonds on the layer of calcite, madder, malachite, and Egyptian blue in part, although it takes longer to achieve the same results as the 1064 nm laser cleaning, and it results in darkening of the malachite and works to blacken the hematite surface after removing the resin stains. However, it is noted to give good results when cleaning resin stains from the surfaces of magnetite and did not result in noticeable color changes.

The results of using wavelengths of 1064 and 532 nm to remove color spots were also proven. The samples gave different results, and it is preferable not to use them in general, as the 532 nm and 1064 nm lasers gave poor results when cleaning the color spots in orange, madder, Egyptian blue, hematite, and magnetite, where the results ranged in the removal of part of the original color layer. With color stains and blackening layers of coloring, it gives good results when removing color spots on calcite surfaces only.

The results of using the 532 nm and 1064 nm lasers have also proven successful in removing microbiological damage stains from the surfaces of calcite, mouth, malachite, Egyptian blue, and magnetite, but the 1064 nm laser is preferred when cleaning the orange, hematite, and gilding layers, as it removes microbiological stains excellently without damaging the original coloring layer, while the 532 nm laser is preferred when cleaning the armpit, as the 1064 nm laser removes the accent layer.

The results of using the laser to remove dust from the surfaces of pigment materials showed the success of the 1064 nm and 532 nm lasers in removing dust from the surfaces of calcite, mouth, malachite, Egyptian blue, and magnetite, while the 1064 nm laser was recommended to clean the gilding layer, orange color, and hematite, as it gives an excellent result when removing dust and dirt without any damage to the colored surface compared to the 532 nm laser, which caused a color change from brown to orange, the blackening of hematite, and also worked to change the color of the gilding layer to a reddish color. By using it on the orpiment layer, the results proved to be more successful than the 1064 nm laser, as it did not cause any color changes.

When using lasers to clean mud stains, it is preferable to start with mechanical cleaning to reduce the thickness of the layer of clay, and then use a laser to remove the remaining light layer of mud. The thickness of the clay (mud) stain was determined by using the wooden steak under the microscope. Then, the samples were exposed to the laser with a wavelength of 1064 nm, and three consecutive pulses were applied to the surface. The results of the cleaning of the clay showed improvement compared to the sample before using the Nd:YAG laser. When the sample was examined using a digital microscope with 50× magnification, the microphotos showed that the pigment layer was not affected by the cleaning operations and no fading process occurred compared to the sample before cleaning. It has also been used to remove surface plankton as well as dust and incoherent dirt on the pigment surface. The 532 nm laser caused discoloration of the mixed orange, hematite, madder, malachite, calcite, and Egyptian blue, and the cleaning process of the mud stain on the golden sheets using the 532 nm laser did not give good results in reducing or removing the stain.

## 4. Conclusions

Laser cleaning, a new surface treatment technology, is applied in many heritage fields to remove the adherent deposits without affecting the original substrates. In this regard, two different wavelengths of a Nd:YAG laser (1064 and 532 nm) were used and evaluated using some examination and analysis techniques such as a microscopy (Dino-lite), X-ray fluorescence (XRF), Fourier transform infrared spectroscopy (FT-IR), and Handy colorimetry to evaluate the effectives of lasers with a wavelength of 532 nm and 1064 nm at removing resin, mud, clay, strange colors, and microbiological stains which distorted the archaeological surfaces. Based on the results obtained in the present study, it was concluded that it is preferable to use lasers at 1064 nm to remove the clay (mud), dust, resin, and microbiological stains. It gave very good results in terms of the degree of color change and the degree of cleaning compared to using the 532 nm laser, which helped clean the pigments without any damage but caused discoloration, blackening, and darkening of surfaces for most of the pigments used in the study. Using these longer wavelength pulses is anticipated to boost the transfer of heat to the bulk material in comparison to using nanosecond 532 nm laser pulses, which can improve the vaporization and re-deposition of the metal. Consequently, greater cleaning efficiencies follow many laser treatments, with superior outcomes at 1064 nm wavelengths as opposed to 532 nm wavelengths.

## Figures and Tables

**Table 1:** The results of the color changes of the standard samples and the aging samples.

Sample	L	A	B	L	A	B	ΔL	ΔA	ΔB	ΔE
	Standard Sample			After Aging			Results			
Orange pigment	56.82	15.08	23.72	52.99	17.79	25.38	−3.83	2.71	1.66	4.98
Calcite	90.65	0.77	8.85	87.29	0.82	12.95	−3.36	0.05	4.1	5.3
Hematite	38.02	12.95	11.42	32.16	12.35	11.30	−5.86	−0.6	−0.12	5.89
Madder	78.62	10.12	12.82	73.13	11.54	14.11	−5.49	1.42	1.29	5.8
Gold layer	76.79	8.38	34.72	71.48	8.08	32.40	−5.31	−0.3	−2.32	5.8
Malachite	55.36	−15.05	16.40	49.98	−17.16	14.89	−5.38	−2.11	−1.51	5.97
Magnetite	28.85	−0.16	3.60	20.79	1.37	3.79	−8.06	1.53	0.19	8.2
Orpiment	72.60	12.91	50.24	64.93	10.06	48.24	−7.67	−2.85	−2	8.4
Egyptian blue	50.48	−6.41	−6.28	35.84	−6.05	−5.80	−14.64	0.36	0.48	14.65

## Data Availability

Not applicable.

## Supplementary Materials

The following supporting information can be downloaded at: https://www.mdpi.com/article/10.3390/mi14071415/s1, Table S1: shows the results of the color change of samples before and after laser cleaning (of the clay stain). Table S2: shows the results of the color change of samples before and after laser cleaning (of the resin stain). Table S3: shows the results of the color change of samples before and after laser cleaning (of the color stain). Table S4: shows the results of the color change of samples before and after laser cleaning (of the microbiological stain (Aspergillus flavus)). Table S5: shows the results of the color change of samples before and after laser cleaning (of the microbiological stain (Aspergillus niger)). Table S6: shows the results of the color change of samples before and after laser cleaning (of the dust strain). Figure S1: The pattern of analysis by X-ray fluorescence of (a) the black pigment of magnetite Fe_3_O_4_, (b) white pigment of Calcite: CaCO_3_, (c) the yellow pigment of Orpiment As_2_S_2_, (d) the red pigment of Hematite Fe_2_O_3_, (e) the orange pigment of orpiment (As_2_S_2_) and hematite (Fe_2_O_3_), (f) the blue pigment of Egyptian Blue CaCuSi_4_O_10_, and (g) the green pigment of Malachite Cu_2_CO_3_(OH)_2_. Figure S2: XRF analysis on the Clay stain on hematite before and after using laser for cleaning. Figure S3: XRF analysis on the resin stain on hematite before and after using laser for cleaning. Figure S4: XRF analysis on the dust stain on hematite before and after using laser for cleaning.
