# Supplementary material for: Comparative Study between First and Second Harmonics of a Nd:YAG Laser for Cleaning Manifestation Damages That Appeared in Pigments Used on Archaeological Cartonnage
Source: Micromachines (Basel). 2023 Jul 13;14(7):1415. doi: 10.3390/mi14071415 (PMC10385187; doi:10.3390/mi14071415)

Supplementary file of

"The effect of Nd-YAG laser wavelengths for cleaning degradation forms on pigments"

1- Results of Handy Colorimeter

a) For cleaning clay stain

Table S1: shows the results of the color change of samples before and after laser cleaning (of the clay stain)

**Sample**		**L**	**A**	**B**	**L**	**A**	**B**	**Δ L**	**Δ A**	**Δ B**	**Δ E**
		Before using laser			After using laser			Results			
Orange pigment	532	47.24	15.59	26.99	42.28	6.33	17.19	-4.96	-9.26	-9.8	14.37
	1064	.2464	.5941	.9952	44.24	13.59	.9952	-2	-1	0	2.2
Calcite	532	90.74	0.49	9.49	63.55	5.14	23.68	-27.19	4.65	14.19	31
	1064	.7419	0.49	.498	90.74	0.49	6.49	-1	0	-2	2.2
Hematite	532	30.00	13.28	11.64	28.14	5.18	5.78	-1.86	-8.1	-5.86	10.17
	1064	.0029	.2821	.6401	8.002	11.28	.6401	-1	-1	0	1.4
Madder	532	65.73	13.47	16.37	60.50	7.98	22.51	-5.23	-5.49	6.14	9.76
	1064	.7346	.4721	.3751	63.73	11.47	14.37	-1	-1	-1	1.7
Gold layer	532	75.85	6.23	31.63	66.03	8.37	35.22	-9.82	2.14	3.59	10.67
	1064	.3715	.523	.6011	50.37	.523	10.60	-1	0	-1	1.4
Malachite	532	46.84	-12.79	15.25	36.44	-15.56	13.81	-10.4	-2.77	-1.44	10.86
	1064	.8454	.791-1	.2541	43.84	-10.79	13.25	-2	1	-1	2.4
Magnetite	532	20.79	1.37	3.79	26.39	1.82	9.91	5.6	0.45	6.12	8.3
	1064	.6419	.610	.812	17.64	.610	0.81	-3	-1	-3	4.3
Orpiment	532	56.74	11.36	45.62	51.83	8.65	31.50	-4.91	-2.71	-14.12	15.19
	1064	.7455	.3601	.6244	54.74	9.36	.6244	-1	-1	0	1.4
Egyptian blue	532	29.72	-4.95	-0.21	37.64	-3.94	4.14	7.92	1.01	4.35	9.09
	1064	.7282	.953-	-0.21	27.72	-2.95	-0.21	-2	2	0	2.8

b) For cleaning resin stain

Table S2: shows the results of the color change of samples before and after laser cleaning (of the resin stain)

Sample		L	A	B	L	A	B	Δ L	Δ A	Δ B	Δ E
		Before using laser			After using laser			Results			
Orange pigment	532	47.24	15.59	26.99	42.28	6.33	17.19	-4.96	-9.26	-9.8	14.37
	1064	47.24	15.59	26.99	45.11	13.19	23.48	-2.13	-2.4	-3.51	4.76
Calcite	532	90.74	0.49	9.49	63.55	5.14	23.68	-27.19	4.65	14.19	31
	1064	90.74	0.49	9.49	57.31	3.98	17.97	-33.43	3.49	8.48	34.67
Hematite	532	30.00	13.28	11.64	28.14	5.18	5.78	-1.86	-8.1	-5.86	10.17
	1064	30.00	13.28	11.64	31.65	11.24	9.75	1.65	-2.04	-1.89	3.2
Madder	532	65.73	13.47	16.37	60.50	7.98	22.51	-5.23	-5.49	6.14	9.76
	1064	65.73	13.47	16.37	55.83	8.52	19.34	-9.9	-4.95	2.97	11.46
Gold layer	532	75.85	6.23	31.63	66.03	8.37	35.22	-9.82	2.14	3.59	10.67
	1064	47.36	3.86	23.91	.5048	6.36	1.14	2.5	2.85	1.14	3.96
Malachite	532	46.84	-12.79	15.25	36.44	-15.56	13.81	-10.4	-2.77	-1.44	10.86
	1064	46.84	-12.79	15.25	29.77	-4.90	8.52	-17.07	7.89	-6.73	19.97
Magnetite	532	20.64	1.61	3.81	.4542	0.40	4.72	3.81	-1.21	0.91	4
	1064	20.64	1.61	3.81	23.18	0.59	2.84	2.54	-1.02	-0.97	2.9
Orpiment	532	56.74	11.36	45.62	51.83	8.65	31.50	-4.91	-2.71	-14.12	15.19
	1064	69.21	9.93	50.36	51.83	8.65	31.50	-17.38	-1.28	-18.86	25.68
Egyptian blue	532	29.72	-4.95	-0.21	37.64	-3.94	4.14	7.92	1.01	4.35	9.09
	1064	29.72	-4.95	-0.21	29.41	-3.16	0.37	-0.31	1.79	0.58	1.9

c) For cleaning color stain

Table S3: shows the results of the color change of samples before and after laser cleaning (of the color stain)

**Sample**		**L**	**A**	**B**	**L**	**A**	**B**	**Δ L**	**Δ A**	**Δ B**	**Δ E**
		Before using laser			After using laser			Results			
Orange pigment	532	47.24	15.59	26.99	35.48	3.17	8.77	-11.76	-12.42	-18.22	24.99
	1064	47.24	15.59	26.99	32.07	9.24	12.29	-15.17	-6.35	-14.7	122.
Calcite	532	90.74	0.49	9.49	50.86	2.07	9.77	-39.88	1.58	0.28	39.9
	1064	90.74	0.49	9.49	50.58	4.20	10.76	-40.16	3.71	1.27	40.35
Hematite	532	30.00	13.28	11.64	34.75	7.20	8.90	4.75	-6.08	-2.74	28.
	1064	30.00	13.28	11.64	29.59	12.68	10.58	-0.41	-0.6	-1.06	31.
Madder	532	65.73	13.47	16.37	43.84	14.22	13.45	-21.89	0.75	-2.92	122.
	1064	65.73	13.47	16.37	32.41	10.43	12.78	-33.32	-3.04	-3.59	33.65
Gold layer	532	73.48	8.08	32.40	29.86	4.36	7.26	-43.62	-3.72	-25.14	50.4
	1064	73.48	8.08	32.40	47.80	6.89	25.54	-25.68	-1.19	-6.86	26.6
Malachite	532	46.84	-12.79	15.25	34.62	-10.77	12.35	-12.22	2.02	-2.9	12.7
	1064	46.84	-12.79	15.25	36.79	-2.51	9.50	-10.05	10.28	-5.75	15.48
Magnetite	532	20.64	1.61	3.81	23.44	4.13	5.60	2.8	2.52	1.79	4.17
	1064	20.64	1.61	3.81	21.72	6.20	6.50	1.08	4.59	2.69	5.4
Orpiment	532	56.74	11.36	45.62	35.28	6.28	17.84	-21.46	-5.08	-27.78	535.
	1064	56.74	11.36	45.62	65.88	7.79	37.55	9.14	-3.57	-8.07	12.7
Egyptian blue	532	29.72	-4.95	-0.21	29.66	0.59	3.01	-0.06	5.54	3.22	6.4
	1064	29.72	-4.95	-0.21	28.62	8.18	7.37	-1.1	13.13	7.58	15.2

d) For cleaning microbiological stain (*Aspergillus flavus*)

Table S4: shows the results of the color change of samples before and after laser cleaning (of the microbiological stain (*Aspergillus flavus*))

**Sample**		**L**	**A**	**B**	**L**	**A**	**B**	**Δ L**	**Δ A**	**Δ B**	**Δ E**
		Before using laser			After using laser			Results			
Orange pigment	532	52.99	17.79	25.38	54.79	4.87	18.6	1.8	-12.92	-6.78	14.7
	1064	47.24	15.59	26.99	43.51	16.92	25.88	-3.73	1.33	-1.11	4.1
Calcite	532	90.74	0.49	9.49	89.17	1.23	11.92	-1.57	0.74	2.43	2.99
	1064	90.74	0.49	9.49	87.16	1.11	11.29	-3.58	0.62	1.8	4
Hematite	532	30.00	13.28	11.64	34.75	7.20	8.90	4.75	-6.08	-2.74	28.
	1064	30.00	13.28	11.64	29.36	14.04	12.96	-0.64	0.76	1.32	1.65
Madder	532	65.73	13.47	16.37	67.10	10.96	16.79	1.37	-2.51	0.42	2.89
	1064	65.73	13.47	16.37	61.59	11.88	15.23	-4.14	-1.59	-1.14	4.6
Gold layer	532	73.48	8.08	32.40	48.42	4.92	23.04	-25.06	-3.16	-9.36	26.9
	1064	73.48	8.08	32.40	71.35	6.42	29.74	-2.13	-1.66	-2.66	3.8
Malachite	532	46.84	-12.79	15.25	43.14	-14.15	12.88	-3.7	-1.36	-2.37	4.6
	1064	46.84	-12.79	15.25	44.42	-10.09	19.82	-2.42	2.7	4.57	5.8
Magnetite	532	20.64	1.61	3.81	22.26	0.92	2.61	1.62	-0.69	-1.2	2.1
	1064	20.64	1.61	3.81	20.84	1.43	4.89	0.2	-0.18	1.08	1.1
Orpiment	532	56.74	11.36	45.62	60.47	12.42	48	3.73	1.06	2.38	4.5
	1064	64.93	10.06	48.24	69.96	9.73	51.29	5.03	-0.33	3.05	5.89
Egyptian blue	532	35.84	-6.05	-5.80	35.00	-5.77	0.56	-0.84	0.28	6.36	6.4
	1064	35.84	-6.05	-5.80	35.01	-4.75	-1.75	-0.83	1.3	4.05	4.3

e) For cleaning microbiological stain (*Aspergillus niger*)

Table S5: shows the results of the color change of samples before and after laser cleaning (of the microbiological stain (*Aspergillus niger*))

Sample		L	A	B	L	A	B	Δ L	Δ A	Δ B	Δ E
		Before using laser			After using laser			Results			
Orange pigment	532	52.99	17.79	25.38	54.79	4.87	18.6	1.8	-12.92	-6.78	14.7
	1064	47.24	15.59	26.99	42.81	14.23	25.09	-4.43	-1.36	-1.9	5
Calcite	532	90.74	0.49	9.49	89.17	1.23	11.92	-1.57	0.74	2.43	2.99
	1064	90.74	0.49	9.49	87.16	1.11	11.29	-3.58	0.62	1.8	4
Hematite	532	30.00	13.28	11.64	23.06	8.63	8.85	-6.94	-4.65	-2.79	8.8
	1064	30.00	13.28	11.64	29.36	14.04	12.96	-0.64	0.76	1.32	1.65
Madder	532	65.73	13.47	16.37	62.66	12.26	15.20	-3.07	-1.21	-1.17	3.5
	1064	65.73	13.47	16.37	65.74	11.77	15.42	0.01	-1.7	-0.95	1.9
Gold layer	532	73.48	8.08	32.40	48.56	5.20	23.65	-24.92	-2.88	-8.75	726.5
	1064	73.48	8.08	32.40	71.35	6.42	29.74	-2.13	-1.66	-2.66	3.8
Malachite	532	49.98	-17.16	14.89	45.7	-15.21	14.91	-4.28	1.95	0.02	4.7
	1064	49.98	-17.16	14.89	44.96	-10.56	12.62	-5.02	6.6	-2.27	68.
Magnetite	532	20.64	1.61	3.81	18.12	2.47	5.03	-2.52	0.86	1.22	2.9
	1064	20.64	1.61	3.81	20.94	1.03	3.50	0.3	-0.58	-0.31	0.7
Orpiment	532	56.74	11.36	45.62	60.47	12.42	48	3.73	1.06	2.38	4.5
	1064	64.93	10.06	48.24	69.96	9.73	51.29	5.03	-0.33	3.05	5.89
Egyptian blue	532	35.84	-6.05	-5.80	35.00	-5.77	0.56	-0.84	0.28	6.36	6.4
	1064	35.84	-6.05	-5.80	34.33	-6.32	-2.86	-1.51	-0.27	2.94	3.3

f) For cleaning dust stain

Table S6: shows the results of the color change of samples before and after laser cleaning (of the dust strain)

**Sample**		**L**	**A**	**B**	**L**	**A**	**B**	**Δ L**	**Δ A**	**Δ B**	**Δ E**
		Before using laser			After using laser			Results			
Orange pigment	532	47.24	15.59	26.99	43.92	12.54	23.75	-3.32	-3.05	-3.24	5.55
	1064	9852.	9617.	8225.	52.99	17.79	25.38	0.1	0.1	0.1	0.17
Calcite	532	87.29	0.82	12.95	84.66	1.06	14.17	-2.63	0.24	1.22	2.9
	1064	9187.	270.	5812.	87.29	0.82	12.95	0.1	0.1	0.1	0.17
Hematite	532	30	13.28	11.64	29.38	7.46	7.22	-0.62	-5.82	-4.42	7.3
	1064	30	13.28	11.64	32.16	12.35	11.30	2.16	-0.93	-0.34	82.3
Madder	532	73.13	11.54	14.11	72.05	9.94	17.28	-1.08	-1.6	3.17	3.7
	1064	3665.	3713.	7216.	70.03	9.81	14.42	4.4	-3.56	-1.85	5.95
Gold layer	532	73.48	8.08	32.40	49.18	5.44	20.89	-24.3	-2.64	-11.51	27
	1064	73.48	8.08	32.40	71.35	6.42	29.74	-2.13	-1.66	-2.66	3.8
Malachite	532	49.98	-17.16	14.89	48.36	-16.64	14.59	-1.62	0.52	-0.3	1.7
	1064	8849.	60-17.	9714.	49.98	-17.16	14.89	0.1	-0.1	0.1	0.17
Magnetite	532	9420.	701.	943.	21.47	1.20	3.25	0.98	0.13	-0.24	1
	1064	9420.	701.	943.	20.79	1.37	3.79	0.3	0.3	0.3	0.5
Orpiment	532	64.93	10.06	48.24	70.21	9.71	50.60	5.28	-0.35	2.36	5.79
	1064	.9336	.069	.2474	64.93	10.06	48.24	1	1	1	1.7
Egyptian blue	532	35.84	-6.05	-5.80	42.27	-8.64	0.70	6.43	-2.59	6.5	9.5
	1064	35.84	-6.05	-5.80	34.33	-6.32	-2.86	-1.51	-0.27	2.94	3.3

2- **Results of XRF elemental analysis of:**

a) The used different pigment

Figure S1: The pattern of analysis by X-ray fluorescence of (a) the black pigment of magnetite Fe_3O_4, (b) white pigment of Calcite: $CaCO_3$, (c) the yellow pigment of Orpiment As_2S_2, (d) the red pigment of Hematite Fe_2O_3 , (e) the orange pigment of orpiment (As_2S_2) and hematite (Fe_2O_3), (f) the blue pigment of Egyptian Blue $CaCuSi_4O_{10}$, and (g) the green pigment of Malachite $Cu_2CO_3(OH)_2$

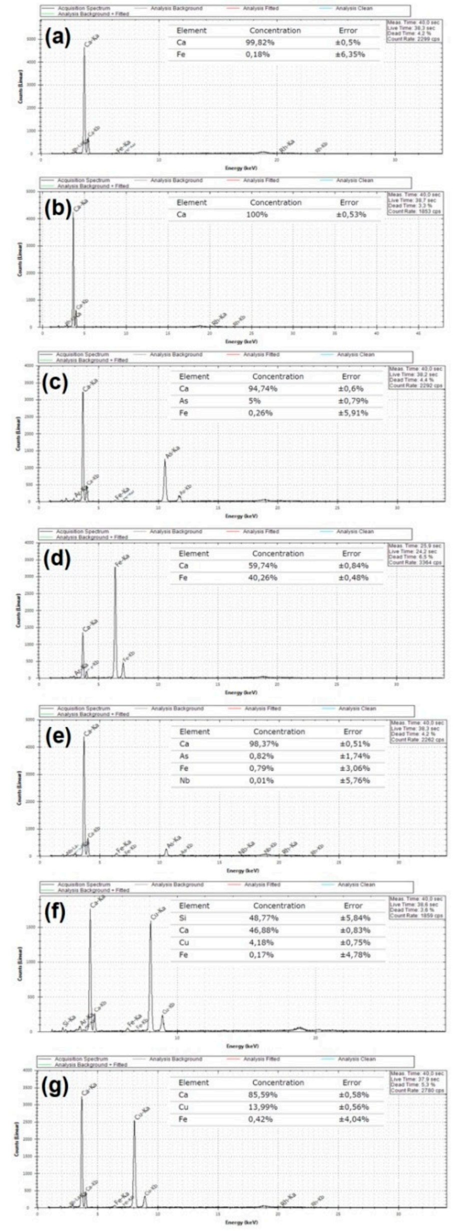

b) The constituent elements of hematite pigments before and after cleaning the stains (clay, resin, and dust) by using 532 and 1064 nm laser cleaning

Figure S2: XRF analysis on the Clay stain on hematite before and after using laser for cleaning

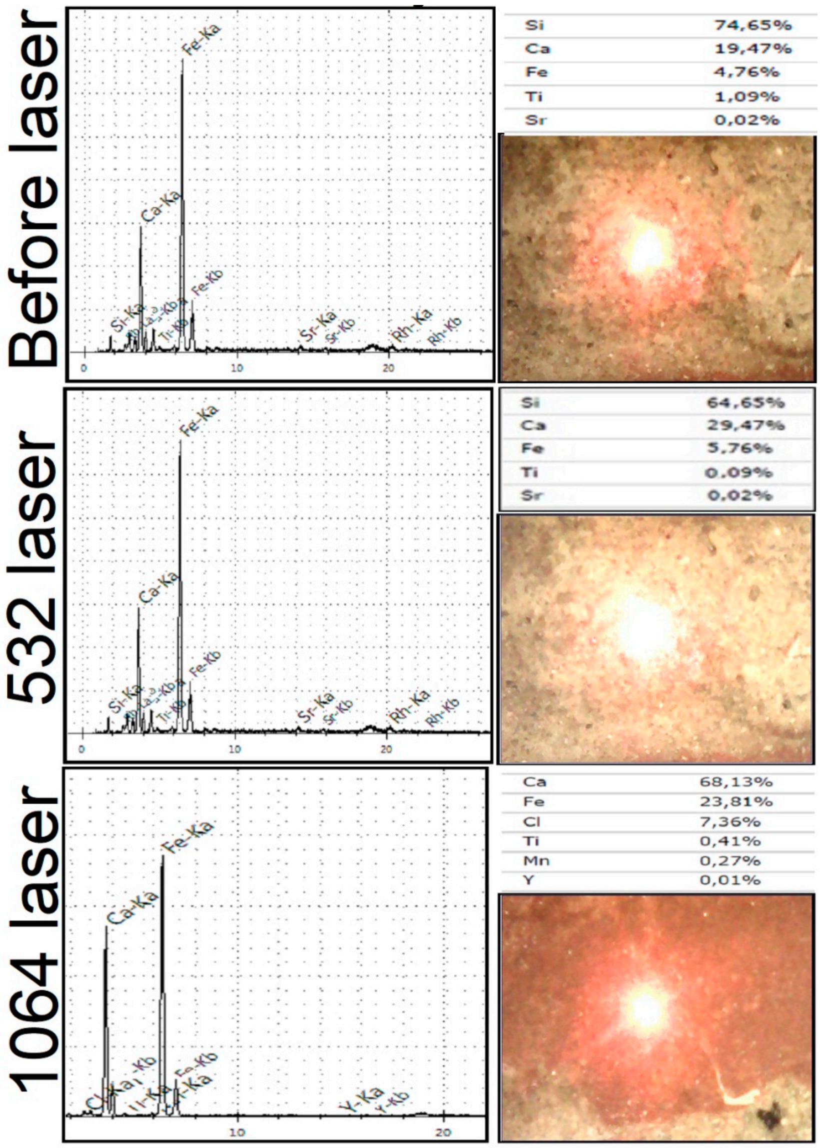

Figure S3: XRF analysis on the resin stain on hematite before and after using laser for cleaning

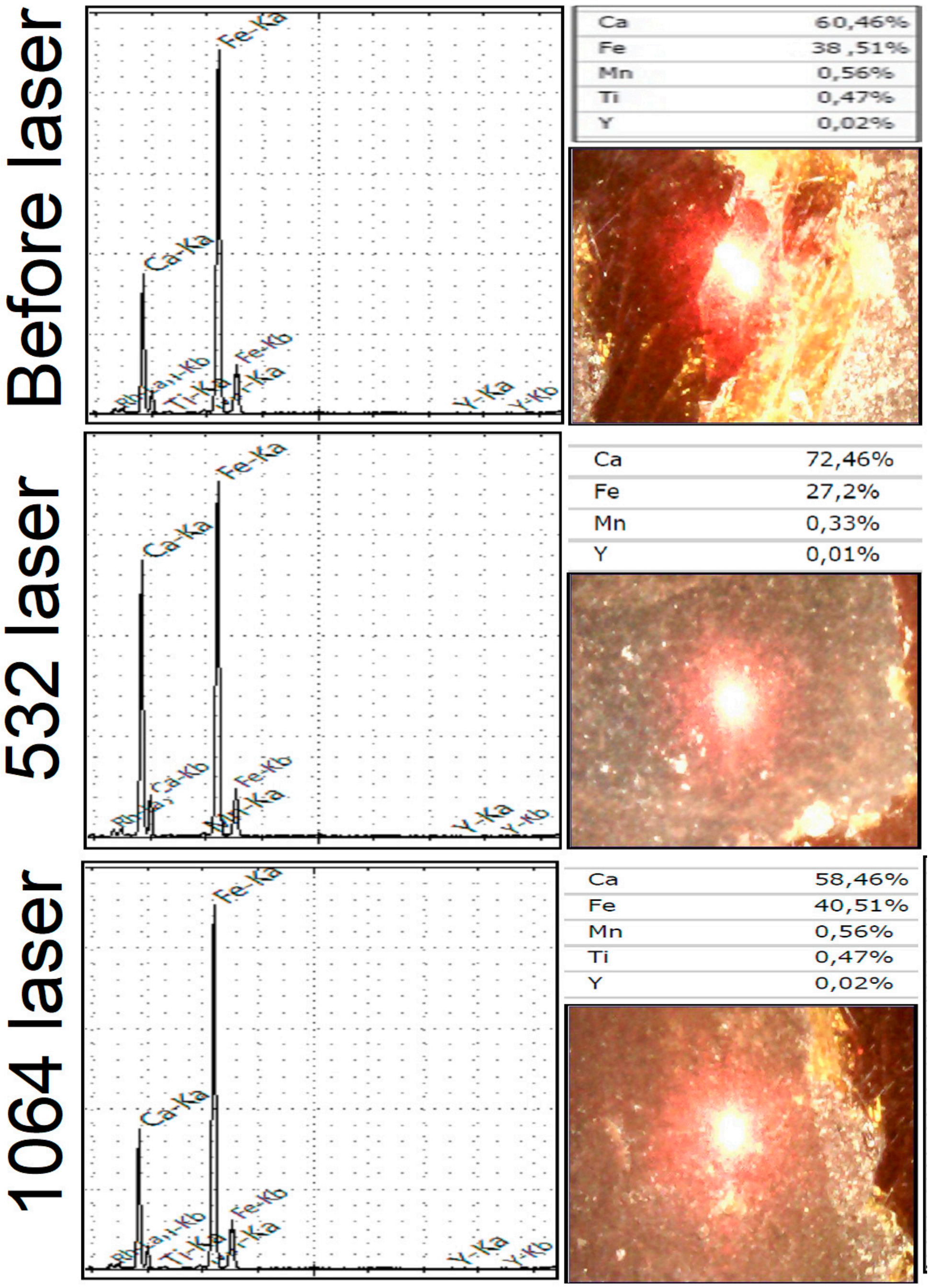

Figure S4: XRF analysis on the dust stain on hematite before and after using laser for cleaning

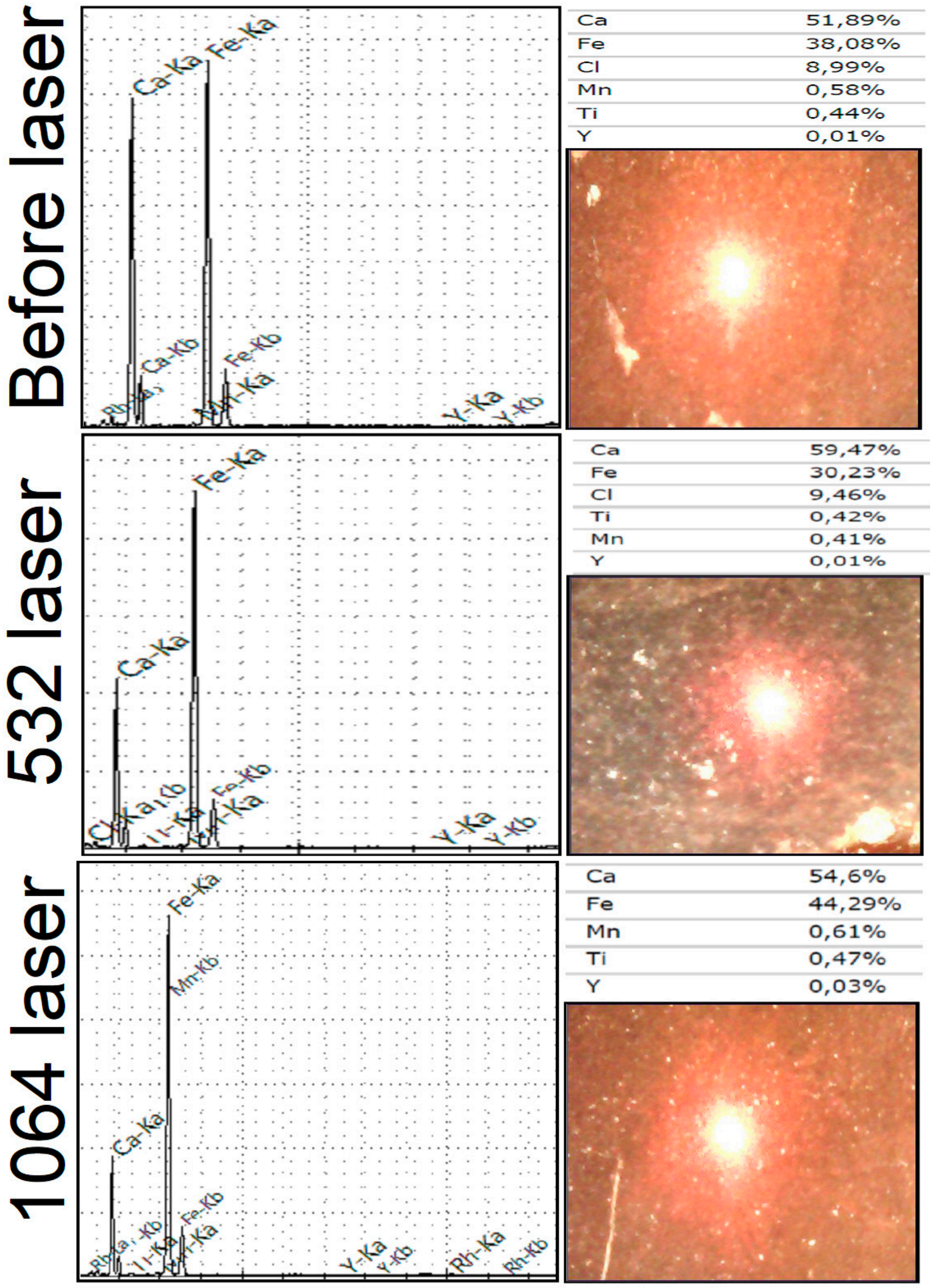

Supplement: Supplementary file 1 [file micromachines-14-01415-s001.zip › micromachines-2483991-supplementary.pdf]
